# Time for a causal systems map of physical activity

**DOI:** 10.2471/BLT.19.236398

**Published:** 2019-01-28

**Authors:** James L Nuzzo, James Steele

**Affiliations:** a Neuroscience Research Australia, Barker Street, Randwick, 2031, New South Wales, Australia.; b Solent University, School of Sport, Health and Social Sciences, Southampton, England.

Regular participation in physical activity is associated with many health benefits. However, about one third of adults worldwide do not achieve recommended levels of physical activity.^1^ Consequently, in recent years, national and international health organizations have developed plans to increase population levels of physical activity.^2^

Recently, Rutter et al. proposed a systems approach to global and national physical activity plans.^3^ To supplement this approach, they developed a systems map with 44 determinants or correlates of physical activity. Numerous lines in their map appear to connect the 44 variables to one another and to physical activity. Rutter et al. state that the map: (i) “provides a visual depiction of how the different parts of a system relate;” (ii) “can help make sense of what otherwise might be perceived as diverse and chaotic relations between large numbers of factors;” and (iii) “can support the development of policy and actions plans to increase physical activity.”^3^

We acknowledge both the introduction of systems theory and the visual depiction of correlates and determinants of physical activity in a figure as initial steps in informing physical activity plans and policies. However, we believe the map by Rutter et al. requires revision or replacement. In its current format, the map cannot inform physical activity policy in a directed manner. In our opinion, the purpose of systems maps should be to reflect causality, meaning, to depict relationships between variables, which, if acted upon, cause predictable changes in physical activity.

We observed several issues with the systems map proposed by Rutter et al. First, we cannot tell what variables are connected by the lines in the map.^3^ A given line sometimes appears to connect one variable to another, while simultaneously travelling underneath additional variables on its way to a third one. If discerning what variables are proposed as being related is not possible, how can the accuracy of the map be evaluated? Second, even when the map seems to show that two variables are connected, the proposed causal direction of the relationship remains unknown, because the lines lack arrows. If discerning what variable is proposed as the cause of another is not feasible, how is it possible to determine what variable to act on with a policy? Third, telling whether a given line is representing a direct effect between two variables or if it is showing an indirect effect mediated through a third variable is impossible. Fourth, lines appear to be missing for some variables that are known to be related, as in the case with age and health status.

Moreover, we believe the proposed systems map is accompanied by a mixed message as to whether it depicts causality. At times, Rutter et al. state explicitly the map is not intended to describe causal relations.^3^ We agree the map does not depict causality. However, we believe such maps should illustrate causality. Importantly, the three papers on system maps referenced to support the proposed systems map of physical activity included causal diagrams with clear connections between variables. Nevertheless, with statements that the map depicts “relations,” “connections” and “interactions,” we believe Rutter et al. imply the map does reflect causality.^3^ In our opinion, such relations, connections and interactions between variables are not helpful in informing policy if they are not causal. Policy is a causal concept,^4^ and therefore attempting to inform policy with a map that does not depict causality is untenable.

## Causal maps

Causal maps, or path diagrams, are illustrations of probable causal pathways between variables. They were developed by Sewall Wright in the 1920s and have gained attention from health, social, and behavioural scientists in the past 20 years.^5^ Causal maps serve a critical role in causal mediation analysis, a framework designed to provide minimally confounded estimates of effects mediated through specific pathways or variables.^5^^,^^6^

One type of causal map is the directed acyclic graph. Directed acyclic graphs overcome the various pitfalls of the proposed systems map. They make model assumptions explicit by showing (i) what variables are proposed as related, (ii) the direction of causation proposed (through arrows on the lines) and (iii) direct and indirect pathways. Moreover, these graphs can handle both linear and nonlinear estimates which should be appealing as systems theory has been adopted as an approach which differs from traditional linear models of cause and effect. Also, causal maps can be used to depict entire systems as well specific domains or relations within a given system.^7^ In fact, the three references cited by Rutter et al. in support of a systems approach each included causal systems maps.^3^

Directed acyclic graphs are developed using rigorous rules,^5^^,^^8^^,^^9^ including in the context of complex systems.^7^ The purpose of these rules is to reduce confounding and help identify causal effects. Free software is available to assist in developing these graphs.^10^

Causal maps for physical activity will not be easy to develop because numerous biological, environmental and intrapersonal factors correlate with or determine physical activity behaviour.^11^^,^^12^ We do not present a causal map for physical activity here because doing so would require an updated systematic review of the literature on all possible correlates and determinants of physical activity, as well as associated confounders and moderators, followed by careful application of the rules of directed acyclic graphs to produce an accurate and informative map. Instead, we recommend national and international health organizations form working groups of exercise scientists and epidemiologists to develop the first causal systems maps of physical activity. We also recommend causal maps become standard features of national and international physical activity plans. Currently, there is little evidence that these plans are effective to increase physical activity at population levels. Nevertheless, if such plans are developed, they should be held to the highest scientific standards, meaning causality and causal diagrams should be featured. We note reports on physical activity, such as the World Health Organization’s *Global action plan on physical activity 2018–2030*, often lack discussions of causality.^2^

Finally, once a causal systems map of physical activity is developed, it does not mean it is irrefutable. Evidence-based assumptions made within the map can be tested with new studies and then modified as new evidence emerges. Thus, a causal systems map of physical activity could help to guide research agendas and policy.

Most researchers in health, social and behavioural sciences aim to understand causal relationships and “predict what will happen to an outcome if the treatment is applied to a group of individuals or if a harmful exposure is removed.”^8^ Clinicians and policy-makers are often interested in making causal inferences from a study’s results.^8^ We therefore disagree that the proposed systems map can “help make sense of what otherwise might be perceived as diverse chaotic relations between large number of factors”^3^ and believe such a map could lead to confusion for policy-makers. Instead, we suggest physical activity plans be informed by scientifically rigorous casual systems maps that are developed through national or international collaboration.
